# Evaluation of Deep Neural Networks for Semantic Segmentation of Prostate in T2W MRI

**DOI:** 10.3390/s20113183

**Published:** 2020-06-03

**Authors:** Zia Khan, Norashikin Yahya, Khaled Alsaih, Syed Saad Azhar Ali, Fabrice Meriaudeau

**Affiliations:** 1 Centre for Intelligent Signal and Imaging Research (CISIR), Department of Electrical and Electronic Engineering, Universiti Teknologi PETRONAS, Seri Iskandar 32610, Malaysia; zia_17004635@utp.edu.my (Z.K.); khaledalsaih@gmail.com (K.A.); saad.azhar@utp.edu.my (S.S.A.A.); 2 ImViA/ITFIM, University of Burgundy, 21078 Dijon, France; fabrice.meriaudeau@u-bourgogne.fr

**Keywords:** encoder–decoder, CNNs, DNN, FCN, SegNet, U-Net, DeepLabV3+

## Abstract

In this paper, we present an evaluation of four encoder–decoder CNNs in the segmentation of the prostate gland in T2W magnetic resonance imaging (MRI) image. The four selected CNNs are FCN, SegNet, U-Net, and DeepLabV3+, which was originally proposed for the segmentation of road scene, biomedical, and natural images. Segmentation of prostate in T2W MRI images is an important step in the automatic diagnosis of prostate cancer to enable better lesion detection and staging of prostate cancer. Therefore, many research efforts have been conducted to improve the segmentation of the prostate gland in MRI images. The main challenges of prostate gland segmentation are blurry prostate boundary and variability in prostate anatomical structure. In this work, we investigated the performance of encoder–decoder CNNs for segmentation of prostate gland in T2W MRI. Image pre-processing techniques including image resizing, center-cropping and intensity normalization are applied to address the issues of inter-patient and inter-scanner variability as well as the issue of dominating background pixels over prostate pixels. In addition, to enrich the network with more data, to increase data variation, and to improve its accuracy, patch extraction and data augmentation are applied prior to training the networks. Furthermore, class weight balancing is used to avoid having biased networks since the number of background pixels is much higher than the prostate pixels. The class imbalance problem is solved by utilizing weighted cross-entropy loss function during the training of the CNN model. The performance of the CNNs is evaluated in terms of the Dice similarity coefficient (DSC) and our experimental results show that patch-wise DeepLabV3+ gives the best performance with DSC equal to 92.8%. This value is the highest DSC score compared to the FCN, SegNet, and U-Net that also competed the recently published state-of-the-art method of prostate segmentation.

## 1. Introduction

According to the report published by the GLOBOCAN 2018 [1], global statistics have indicated that prostate cancer is the second most leading diagnosed cancer in men, after lung cancer. In 2018 alone, more than 1.2 million new cases of prostate cancer were reported worldwide with higher cases in developed countries. Prostate cancer may be asymptomatic in the early stage and often has an indolent course, and symptoms may not appear until the cancer is in its more advanced stages [2]. Hence, early detection is highly critical in preventing further growth of cancerous cells in the prostate gland.

In the current clinical approach, the prostate-specific antigen (PSA) blood test and transrectal ultrasound (TRUS) biopsy are applied together for the diagnosis of prostate cancer [3]. However, PSA testing has low specificity because men without cancer have also been identified with elevated PSA and many patients with cancer did not have elevated levels of PSA [4]. On the other hand, TRUS biopsy suffers from low sensitivity due to unclear optimal threshold for biopsy [5]. Due to the low specificity of PSA and low sensitivity of TRUS biopsy, these tests have restricted diagnostic value [6].

Machine learning approaches in image-based diagnosis, disease prognosis, and risk assessment are becoming increasingly successful. For the diagnosis of prostate cancer, magnetic resonance imaging (MRI) is a common modality for evaluation of prostate cancer, providing superior anatomic or pathologic visualization when compared with ultrasound and computed tomography (CT). In the past few years, the use of MRI was proposed by the prostate cancer research community as the most accurate non-invasive screening tool for prostate cancer diagnosis, staging [7], disease monitoring, and treatment management [8].

The prostate gland can be divided into three zones, peripheral zone (PZ), transitional zone (TZ), and the central zone (CZ). Having the advantages of good resolution and contrast in soft tissues, T2W MRI is used to differentiate the different zones of the prostate gland [9]. It was established that the ratio of developing prostate cancer in the prostate gland varies according to the zone. PZ has the highest rate of 70–80%, whereas the TZ rate is 10–20%, while, in CZ, the rate is only 5% [10]. In a typical examination of MRI images, the radiologist performs slice by slice visual inspection and prostate segmentation which can be time-consuming especially for large-scale samples. In addition, the result of manual segmentation varies depending on the skill of the expertise [11]. Hence, to improve the diagnosis of prostate cancer, there is a need for an automatic segmentation technique that is robust to variability in shape, size, image intensity around the gland and surrounding regions, anisotropic spatial resolution, and scanner type [12,13].

In this article, we evaluated and compared the performance of four encoder–decoder CNNs; FCN, SegNet, DeepLabV3+ and U-Net in segmenting prostate T2W MRI into whole gland (WG), or into three zones PZ, TZ, and CZ depending on availability of dataset label. The training and testing of the CNNs are conducted with selected image pre-processing techniques, data augmentation techniques and selected loss function for dealing with the issue of class imbalance.

The paper is outlined as follows. In Section 2, we reviewed papers related to image segmentation focusing on atlas-based segmentation, model-based segmentation, deep learning-based segmentation, and patch CNN-based segmentation. This section also includes a summary of our contribution. Next, Section 3 presents materials and methods, and Section 4 presents results and discussion. Lastly, Section 5 concludes the paper and suggests further work in this area.

## 2. Related Work

Prostate segmentation, a necessary step in automatic image analysis, involves delineating the prostate boundaries. Segmentation will limit subsequent image processing only on the organ of interest. This section highlights some important works on prostate segmentation based on deformable models, and region-based and patch-based CNNs.

Before the breakthrough of deep learning, segmentation based on deformable models [14,15], hybrid methods [16,17,18,19], and atlas-based [20,21,22,23] were introduced. In principle, the deformable models were implemented as the physical bodies with certain elasticity and force properties that try to keep their form, while the image to be segmented was represented as a potential field with the force that tries to stretch the model and adapt to it. To achieve this, deformable-based segmentation incorporates knowledge about the shape, object orientation, continuity, elasticity, or smoothness of the region to be segmented.

In atlas-based segmentation, two major steps are atlas construction and atlas registration where the prior knowledge related to size, orientation, or shape needs to be incorporated [20,21,22,23]. The three basic building blocks for atlas registration are image transformation, similarity measure, and optimization algorithm that finds the maximum similarity measure between atlas and target region.

Deformable-based methods are sensitive to initialization because the contour is driven by a pre-defined energy function in a variational manner, giving local convergence. As a result, the shape of the object may not be well preserved, especially in the presence of image noise and in the non-homogeneous intensity region [14,15]. On the other hand, atlas-based methods lack the flexibility in local tuning the segmentation boundary.

Martin et al. [17] combined the deformable model and probabilistic atlas-based model for the segmentation of prostate MRI achieving the median Dice similarity coefficient (DSC) score of 0.86. Zhang et al. [18] proposed a method that uses a clustering method to separate the prostate gland from the surrounding tissues followed by a postprocessing phase through active contours, giving an overall accuracy with mean DSC of 0.84 ± 0.04.

Methods based on deep learning had shown remarkable results relative to the combination of deformable model and atlas-based segmentation. Hence, various methods were proposed to exploit the advantages of both methods while avoiding their respective drawbacks, including combining both deformable models and atlas-based models. In [16], Cheng et al. combined a supervised atlas-based active appearance model (AAM) and CNN was achieving DSC value equal to 0.925. The method utilized the volume of interest (VOI) generated by AAM and later inputs to CNN as learnable features, and this has increased the overall accuracy of prostate segmentation. Yanrong Guo et al. [19] presented a novel hybrid model by combining a deep learning model and deformable model where the features extracted by the deep learning model were matched to the atlas map by a sparse matching method. This method has resulted in DSC value of 87.1%±4.2%. The subsequent paragraphs highlight some semantic segmentation methods based on deep learning styles.

Long et al. [24] introduced a fully convolutional neural network (FCN) for scene segmentation tasks. The FCN is built from a classical CNN but only consists of locally connected layers including convolution, pooling, and upsampling, whereby the last fully connected layer is replaced by another broad receptive field convolution layer. Since the architecture does not include fully connected layers, the number of parameters and computation time is reduced. In addition, unlike the traditional CNN model, the network can work with any image size without requiring any fixed number of units at any stage, as all connections are local.

The application of CNN for the segmentation of prostate MRI was investigated in [25]. The network, known as PSNet is a fine-tuning of the FCN model proposed by Long et al. [24] trained on the PASCALVOC data set [26] and retrained for the prostate segmentation. Specifically, PSNet fine-tuned the last three layers of the FCN because these layers learn high-level specific features that are related to the prostate gland [27,28].

In [29], Ronneberger et al. proposed U-Net model, which is proposed for medical imaging segmentation tasks. The U-Net model concatenates the contracting side (encoder) and expanding side (decoder) to compensate the loss of spatial resolution due to the max pooling operations. In another work by Milletari et al. [30], the Dice loss function was used to solve the problem of class imbalance between background and foreground pixels and obtained accurate 3D segmentation of the prostate T2W MRI using U-Net architecture. In the work by Yu et al. [31], residual connections are incorporated into the convolutional and deconvolutional part of U-Net and the proposed architecture won the first place in 2017 Computer-Assisted Intervention Conference using the PROMISE12 challenge dataset.

In recent work by Rundo et al. [32], a new segmentation method based on U-Net was proposed. The CNN network known as Use-Net introduced Squeeze-and-Excitation (SE) blocks in both encoder and the decoder of U-Net architecture. The network was trained and tested on multi-dataset allowing for both intra and cross dataset generalization. Their findings revealed that the SE blocks enable adaptive feature recalibration, hence, providing excellent cross-dataset generalization.

Patch-based CNN segmentation methods were proven to result in better pixel-wise segmentation of biomedical images [33,34,35,36,37,38,39]. In [33], Zhang et al. applied patch-based CNN for segmentation of the multi-modality MRI brain dataset, T1, T2, and fractional anisotropy (FA) MRI. The brain region was segmented into white matter, grey matter, and cerebrospinal fluid. For each case, more than 1K patches were extracted at four different sizes; 9×9, 13×13, 17×17 and 22×22 for training and testing.

In [34], Tajbakhsh et al. demonstrated that fine-tuning of a pre-trained CNN in a layer-wise manner leads to incremental performance improvement in classification, detection, and segmentation. The performance evaluation was made for three different medical imaging modalities; polyp detection in colonoscopy images, pulmonary embolism detection in CT pulmonary angiography, and intima-media boundary segmentation from the ultrasound of carotid intima-media thickness. The training set comprised of stratified image patches with an equal number of positive and negative classes. In another work by Kooi et al. [37], automatic detection of breast lesion in mammography was investigated using 39,872 patches of size 250×250 for training the CNN model.

In this article, we evaluated the performance of four CNNs; FCN, SegNet, DeepLabV3+, and U-Net for segmentation of prostate T2W MRI trained in a patch-wise framework. The four CNNs are selected as they have given excellent performance in other applications including road scene, biomedical image, and natural image segmentation. Our contribution in this work includes a head-to-head comparison of the four CNNs, trained in the best possible framework using selected image pre-processing techniques, methods for increasing the number of images and its variation, and a method to tackle the issue of class imbalance. In particular, image resizing, center-cropping, and intensity normalization are applied for addressing the issues of inter-patient and inter-scanner variability as well as the issue of dominating background pixel over a prostate pixel. Issues related to small training images are addressed by using patch extraction while data augmentation is used to increase the variation in images and has improved the network accuracy. In addition, the class imbalance issue was addressed using the weighted cross-entropy loss function during network training. The selected methodology is proven to improve the segmentation accuracy measured in terms of DSC values.

## 3. Material and Methods

**Figure 1:**
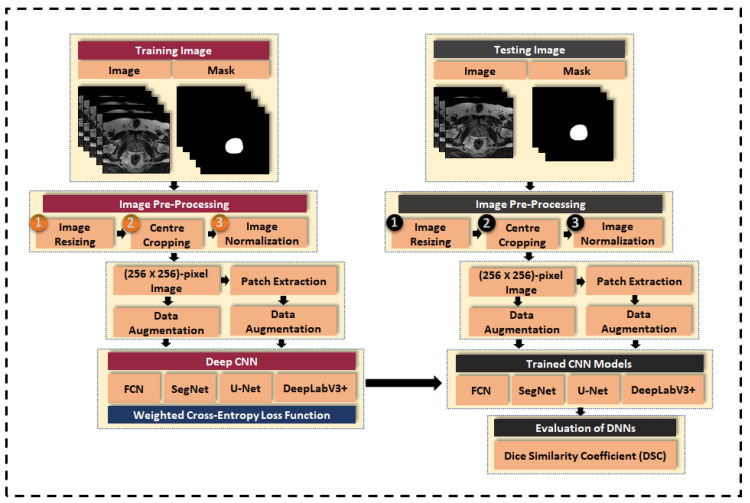
General methodology for evaluation of encoder–decoder CNN architectures in semantic segmentation of prostate T2W MRI.

In this section, we cover the methodology for training and testing of the CNNs in segmenting the whole prostate gland, peripheral zone, and central gland from T2W MRI of two different datasets. Firstly, a brief description of the four CNNs architecture is discussed. Secondly, the details of the two datasets used in this work are described, covering the scanner types, image dimensions, and class labels. In the third section, methods for image pre-processing, data augmentation, and patch extraction are discussed. Next, the technique of class balancing is presented as a measure to prevent the network from being biased toward classes with a larger number of pixels. Then, the weighted cross-entropy loss function used in the optimization of the network parameters is presented. Lastly, the Dice similarity coefficient (DSC) is presented as the performance metric for evaluating the CNN-based segmentation methods. The general framework for evaluation of the CNNs is illustrated in Figure 1.

### 3.1. Deep CNN Architectures for Prostate Segmentation

The following deep CNN models which follow encoder–decoder structure are selected for comparative performance evaluation of semantic segmentation of prostate T2W MRI; FCN [24], U-Net [29], SegNet [40], and DeepLabV3+ [41]. A brief description of each CNN models is presented in the subsequent paragraphs.

Fully convolutional neural network (FCNs) [24,42,43] is based on existing image classification network such as AlexNet [44], VGG16 [45], and GoogleNet [46] to serve as the encoder module (downsampling path) and appended with a decoder module (upsampling path) with transposed convolutional layers. The encoder module is built from local connected layers such as convolution, pooling, and upsampling without any dense layer in the architecture. Since all connections are local, the network can work with any image size. The downsampling path and upsampling path will respectively capture both the context and localization or spatial information. However, pooling or downsampling will result in loss of spatial information. Skip connections are used to recover the spatial information in downsampling layers, hence combining context information with spatial information. Specifically, the skip connections concatenate the feature map from downsampling with upsampling layers at different levels of resolution.

Originally developed for biomedical image segmentation, U-Net is a variant of FCN that was modified to work on a few training images giving better segmentation performance [29,47]. The network has a U-shaped architecture that consists of an encoder–decoder network and interconnected by skip connections. The encoder extracts the spatial feature map from the input image using the convolutional layer, while the decoder performs the upsampling process to get segmented output from the encoder feature map. In feature map construction, a 3×3 convolution operations are performed, followed by stride of two 2×2 max pooling. This process consists of four convolutional operations and, after each downsampling, the number of filters is doubled in the next convolutional layer. The output feature map of 3×3 convolution operation is forwarded to the decoder part which is upsampled using 2×2 transposed convolution operation, reducing the feature map by half. The upsampling stage and two convolution operations are repeated four times, while at each stage the numbers of filters are halved. The information of feature maps from the convolutional operation of an encoder is transferred to the decoder before the pooling operation. The skip connections between the encoder–decoder networks are used to recover the information lost from the pooling operation. The final segmented output is obtained by using a 1×1 convolution operation on the upsampled feature map. All convolutional layers in U-Net used rectified linear unit (ReLU) as the activation function except for the final convolution layer which used sigmoid activation function.

SegNet is a pixel-wise semantic segmentation technique originally developed for outdoor and indoor scene understanding [40,48]. The architecture consists of encoders using only forward connections from VGG architecture, non-trainable layers, and a corresponding set of decoders followed by a pixel-wise classifier. With no fully connected layer, this had reduced the network parameters from 134 M to 14.7 M, which makes it suitable for computationally efficient embedded systems. If the classification network is directly adopted for pixel-wise segmentation, the segmentation result will be poor. This is resulted from the max pooling and subsampling operations that reduce the feature map resolution and hence reduce the output resolution. To maintain the same output image resolution as the input image, the low-resolution feature maps are upsampled by SegNet using memorized pooling indices. This enables the network to store boundary information and reduce the number of trainable parameters.

DeepLabV3+ is another CNN developed for semantic segmentation that is comprised of the encoder–decoder network [41,49,50]. The highlights of the architecture are the use of atrous or dilated convolution and atrous spatial pyramid pooling (ASPP) [51]. To maintain the spatial resolution of the output feature map, DeepLabV3+ used the Xception model as their backbone and made use of depth-wise separable convolution in place of max pooling layers. Instead of using standard convolution, depth-wise separable convolution is used which segregate the operation into depth-wise and point convolution. Feature maps are generated from each input channel by depth wise convolution which applies separable convolution with (r−1) zeros being placed within the successive filters. The output of a depth wise convolution is accumulated by utilizing point wise convolution. In the decoder part, the encoder feature map is upsampled by a factor of 4 and then concatenated with low-level features. Here, the 1×1 convolution is applied for achieving reduction in the number of channels. Lastly, 3×3 convolution is applied on a feature map and subsequently being upsampling by factor of 4 to get the output having equal size to the input.

### 3.2. MRI Dataset

Two MRI datasets are used for evaluation of the CNN architectures, from (1) NCI-ISBI 2013 challenge and (2) Universiti Kebangsaan Malaysia Medical Centre (UKMMC). The summary of the two datasets is as follows:

1. NCI-ISBI 2013 is a prostate 3T collection of 40 patients having 542 T2-weighted MR slices available at the Cancer Imaging Archive (TCIA) site [52]. The images were collected at Radboud University Medical Center, Netherlands, using Siemens TIM 3 Tesla scanners [53]. This dataset has three different image sizes, 384×384, 320×320 and 256×256, having a thickness of 4 mm. The image masks are labeled to 3-class, peripheral zone (PZ), Central Gland (CG), and background.
2. UKMMC dataset is a prostate 3T collection consists of 11 patients having 229 T2-weighted MR slices that are collected from UKMMC. The images were acquired using a 3 Tesla Siemens TIM MRI scanner with a surface coil. The image dimensions are 384×384 and 320×320, with a thickness of 3 mm. The image masks are labeled to 2-class, whole prostate gland (WG), and background.

### 3.3. Image Pre-Processing

Since the two datasets used in this work, NCI-ISBI 2013 and UKMMC, were obtained using two different scanners, there is variation in terms of image dimensions and resolutions. Therefore, image normalization is necessary in order to work with datasets having a common dimension while reducing both inter-patient and inter-scan variability. Details on the pre-processing steps are given in the subsequent paragraphs.

#### 3.3.1. Image Resizing

The images of NCI-ISBI 2013 dataset come in three sizes, 384×384, 320×320 and 256×256, whereas, in the UKMMC dataset, the images come in two sizes, 384×384 and 320×320. Therefore, in order to achieve common image size, image resizing is performed by scaling the images to a size of (320×320)-pixel using the nearest-neighbor interpolation method [54].

#### 3.3.2. Center-Cropping

**Figure 2:**
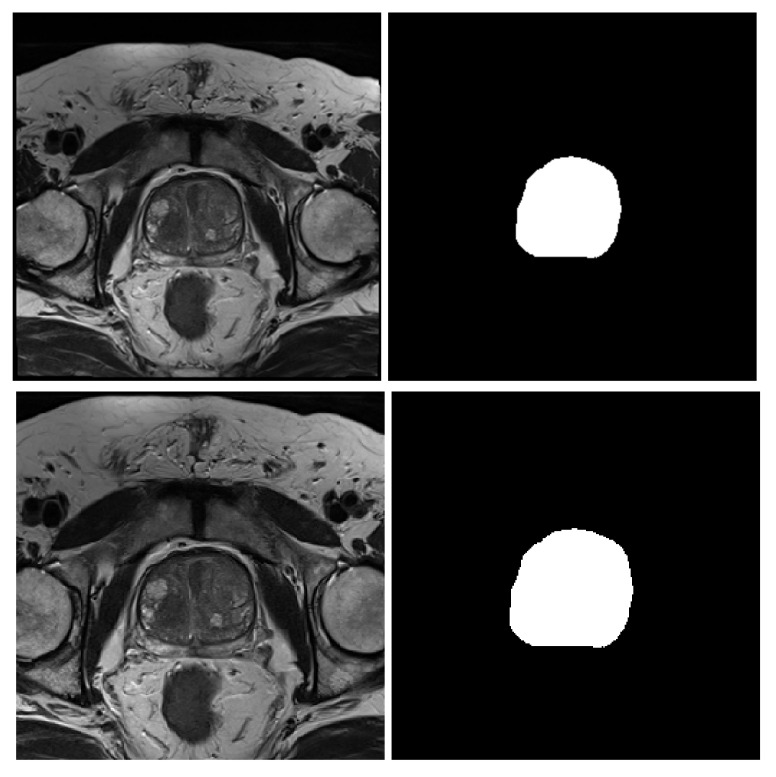
Center-cropping of MRI prostate image, original MRI (**top-left**), center-cropped image (**bottom-left**), and the corresponding masks (**top/bottom-right**).

The size of the prostate gland in the MRI scan is relatively small compared to the background, and this will result in background pixels to dominate over the prostate pixel resulting in poor prostate segmentation. Hence, center-cropping is necessary for reducing the number of background pixels [55,56], and to reduce the computation time with redundant pixels. Since the output size from image resizing is (320×320)-pixels, the dimensions of these images are center cropped to (256×256)-pixel. A sample of the center cropped image and its corresponding mask is shown in Figure 2.

#### 3.3.3. Intensity Normalization

Intensity normalization is used to reduce variation in the intensity distribution of images in one dataset which occurs due to inter-patient variability [57]. This will also improve the comparability of the images across different subjects and give better segmentation results. Hence, intensity normalization is applied to both datasets.

### 3.4. Data Augmentation

During the training of deep neural networks, data augmentation is performed to improve the accuracy of the training networks. Data augmentation will essentially increase the spatial variation of the images and improve the network accuracy [58]. The two data augmentation techniques used are random reflection and translation by ± 10 pixels in the *x*-axis and *y*-axis directions.

### 3.5. Patch Extraction

Training DNNs for accurate prostate segmentation requires large datasets, hence we used a patch-wise framework. The four different encoder/decoder networks are evaluated in terms of DSC for different sizes of patches, including 64×64, 128×128, and 192×192, similar to the work of Sekou et al. [59]. It is observed that the performance of the CNNs decreases in the smaller patch sizes because of the small prostate region compared to the background region. In smaller patch sizes of 64×64 and 128×128, the prediction of the prostate gland is lower than in higher patch size of 192×192. This explains why the CNN architectures will result in better segmentation in the case of (192×192)-patch than the (64×64)-patch and (128×128)-patch.

**Figure 3:**
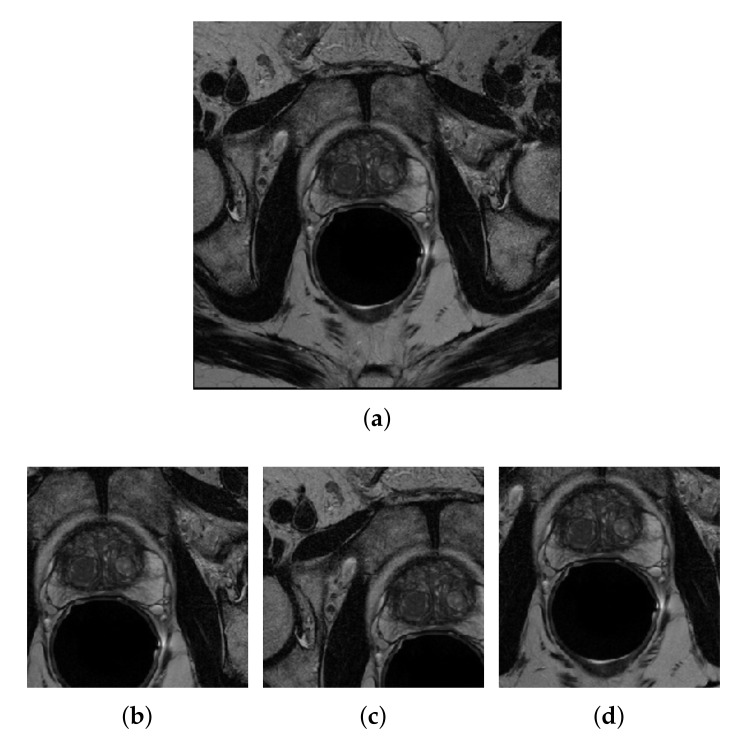
Sample of three (192×192)-pixel patches (**b–d**) generated from the (256×256)-pixel image in (**a**).

In our case, from each MRI slices, 20 and 30 (192×192)-pixel random patches were generated from NCI-ISBI 2013 and UKMMC dataset, respectively. For NCI-ISBI 2013, 460 images were selected for training from a total of 542 MRI slices, giving 9200 images, a 20-fold increase in the number of training images. On the other hand, since the UKMMC is a relatively small dataset, more patches are needed to be generated from this dataset. From 176 MRI slices, patch extraction has generated 5280 total training images, which is a 30-fold increase from the original 176 slices. A sample of patches generated from one of the datasets is shown in Figure 3.

### 3.6. Class Weight Balancing

It is common in medical volume scan that the anatomy of interest occupies only a small region of the scan, like in the case of prostate MRI. As a result, the number of pixels for image background is higher than the prostate gland, resulting in imbalance class labels. Specifically, for the NCI-ISBI 2013 dataset, the ratio of pixels for background to central gland and peripheral zone is 95.5:1.9:2.6, whereas, in the UKMMC dataset, the ratio of background to whole gland is at 94.94:5.06. Class imbalance can be detrimental for the learning process as the network learns more on dominant classes and biases towards these dominant classes. To improve the learning process and avoid being trapped in local minima, the class weighting approach can be used as a systematic way to improve the low dominant class [60]. To counteract class imbalance present in the dataset, larger weights are assigned for labels with less total pixels and smaller weights for labels with more total pixels.

If the number of pixels in a particular class is denoted as $N_{c}$, where *c* corresponds to background, CG and PZ for 3-class dataset, and *c* corresponds to background and WG for 2-class dataset. Hence, $N_{c}$ is (1×3)-array and (1×2)-array for 3-class dataset and 2-class dataset, respectively. Let *T* represent the total number of pixels in the images, then the (1×2) or (1×3)-array of image frequency, $F_{c}$, of a class is the ratio of $N_{c}$ to *T* given by

(1) $$F_{c}=\frac{N_{c}}{T}.$$

From here, the (1×2) or (1×3)-array of class weight, $W_{c}$, for a set of training data can be calculated by finding the ratio of median of $F_{c}$ to $F_{c}$

(2) $$W_{c}=\frac{median(F_{c})}{F_{c}}.$$

**Figure 4:**
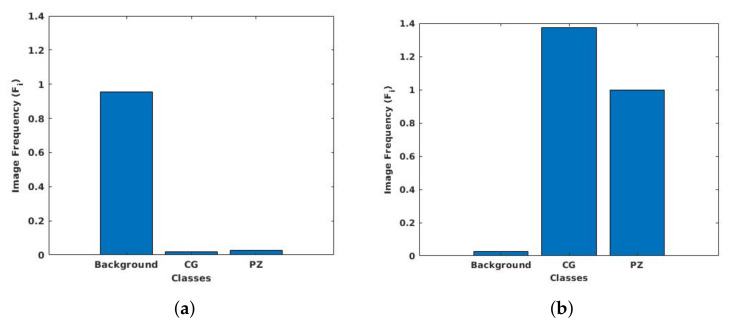
Pixel distribution of (**a**) original images and (**b**) after class weighting for the NCI-ISBI 2013 dataset.

**Figure 5:**
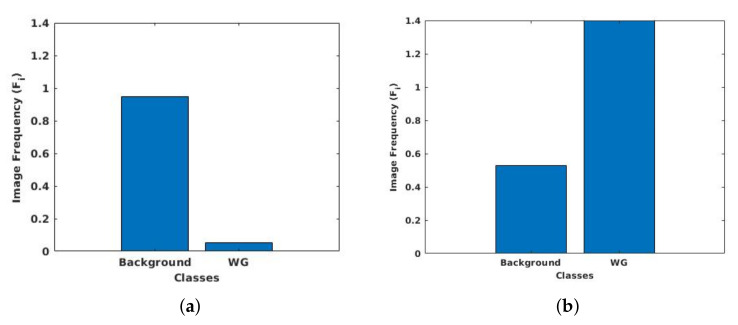
Pixel distribution of (**a**) original images and (**b**) after class weighting for the UKMMC dataset.

Histograms of pixel distribution of 3-class NCI-ISBI 2013 dataset and 2-class UKMMC dataset, before and after applying class weighting approach, are shown respectively in Figure 4 and Figure 5.

### 3.7. Loss Function

In this work, the weighted cross-entropy (WCE) loss function is selected for the CNNs because it helps the network to better differentiate between background and prostate pixels, and is especially handy when there is class imbalance. The WCE loss function penalizes each class based on the median frequency of each class, which is formulated as follows [61]:

(3) $$WCE=-\frac{1}{n}\sum_{i=1}^{n}W_{c,i}\left[T_{i}\log P_{i}+\left(1-T_{i}\right)\log\left(1-P_{i}\right)\right],$$

where the sum is run over all training images, *n*. The variable $P_{i}$ is the predicted segmentation class, $T_{i}$ is the target or the ground truth segmentation label, and $W_{c,i}$ is the class weight calculated from Equation (2).

### 3.8. Evaluation Metric

The performance of the CNNs for segmentation of prostate T2W MRI is evaluated based on intersection between *target* and *prediction* sets using dice similarity coefficient (DSC) [62]. In essence, DSC measures the resemblance of elements between *prediction* and *target* sets given as

(4) $$DSC=2\frac{\left|T⋂P\right|}{\left|T|+|P\right|}\times 100,$$

where |T| and |P| represent the number of elements in target and prediction sets, respectively. The DSC values range between 0 to 1, with 1 indicating the best-case scenario.

## 4. Results and Discussion

### 4.1. Experimental Set-up

The code for training and testing the CNNs model is developed on the MATLAB 2019b platform, using a PC equipped with 32 GB memory, Intel Core i7-8700k CPU, GeForce RTX 2080Ti graphic card with 32 GB memory and 10.1 Cuda edition. During the training, the network weight is updated by the Adam optimizer [63] with learning rate selected at 0.0001 and minibatch size equal to 16. Using patch extraction, total training images generated from NCI-ISBI 2013 and UKMMC dataset are 9200 and 5280, respectively. To ensure that the trained CNNs are well generalized, 5-fold cross validation strategy is utilized [64]. Here, the data are divided into 80% for training and 20% for testing the networks [64,65]. The performance of the CNNs is obtained from an average value of the 5-fold cross-validation.

### 4.2. Quantitative and Qualitative Performance

The segmentation performance by the CNNs is quantitatively evaluated in terms of DSC values, averaged over all test images. The DSC values for datasets NCI-ISBI 2013 and UKMMC are shown in Table 1 and Table 2, respectively. The results are presented for both without patch extraction and with patch extraction. Subsequent paragraphs provide an analysis of segmentation results for both datasets.

The encoder–decoder CNNs are trained and tested in two scenarios: (1) without patch extraction using (256×256)-pixel input images and (2) patch-wise extraction using (192×192)-pixel patch size input images. The DSC values for NCI-ISBI 2013 dataset for FCN, SegNet, U-Net, and DeepLabV3+ are presented in Table 1. The NCI-ISBI 2013 is a 3-class labeled data where the prostate regions are divided into two regions, peripheral zone (PZ) and central gland (CG). The DSC values in Table 1 indicate that the patch-wise method will result in better segmentation mainly because it has increased the amount of training data hence, enriching the deep neural network with more data. In particular, using patch-wise implementation, average DSC gain, calculated from the four CNNs is 2.5% and 2.1% for PZ and CG, respectively. In addition, the results clearly show that DeepLabV3+ gives the best prostate segmentation with the highest DSC values of 78.9% and 92.8% for PZ and CG, respectively. In general, the better performance for the segmentation of CG compared to PZ by the CNNs is due to the higher ratio of CG volume to PZ volume in the NCI-ISBI 2013 dataset.

**Figure 6:**
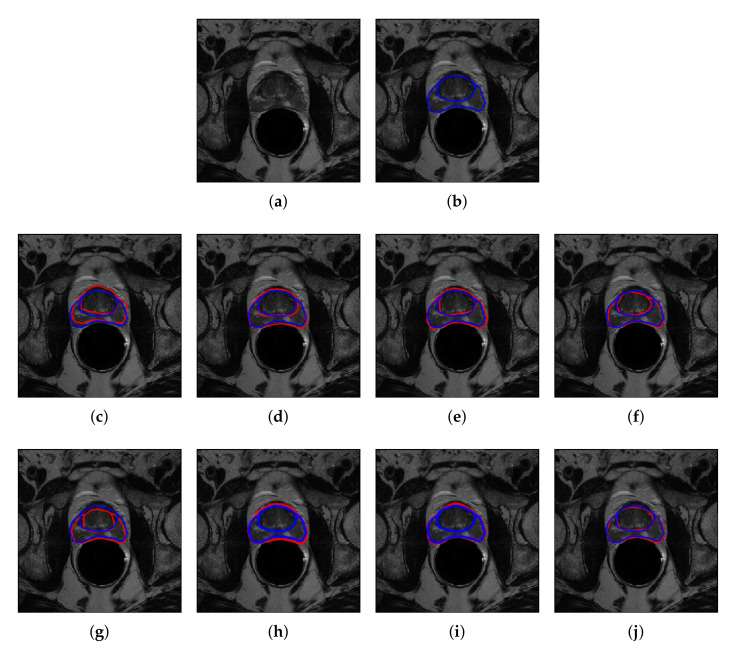
Segmentation of prostate T2W MRI, from NCI-ISBI 2013 dataset into PZ and CG regions. (**a**) Original image, (**b**) ground truth boundary, where predicted boundary (red), overlay on the ground truth (blue), based on the method without patch extraction using, (**c**) FCN, (**d**) SegNet, (**e**) U-Net and (**f**) DeepLabV3+, and based on patch extraction using (**g**) FCN, (**h**) SegNet (**i**) U-Net and (**j**) DeepLabV3+.

As for qualitative analysis, we present a sample of segmented images as shown in Figure 6 for both without and with the patch-wise method for PZ and CG regions. Visual comparison between without patch and with patch-wise indicates that more accurate segmentation can be achieved by patch-wise method. This highlights the significance of patch extraction which has enriched the CNNs with more data and improves its accuracy. It is notable from Figure 6 that the mask generated by the best CNN, DeepLabV3+ is closely similar in shape and size to the ground truth mask. In addition, DeepLabV3+ can accurately segment the region around the prostate boundary. On the other hand, FCN, SegNet, and U-Net are also able to segment prostate into PZ and CG regions but with less accurate segmentation, especially around the pixels close to the prostate boundary.

**Figure 7:**
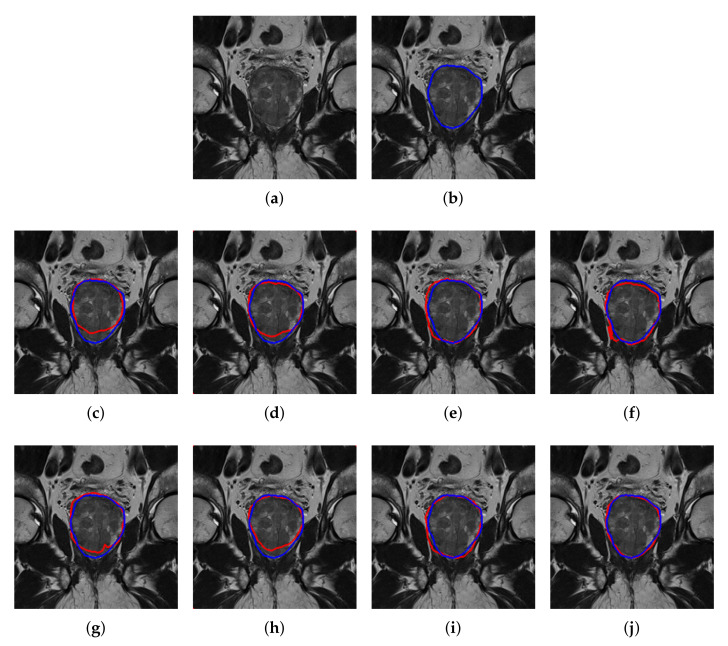
Semantic segmentation of WG prostate T2W MRI, from UKMMC dataset. (**a**) Original image, (**b**) ground truth boundary where predicted boundary (red), overlay on the ground truth (blue), based on the method without patch extraction using, (**c**) FCN, (**d**) SegNet, (**e**) U-Net and (**f**) DeepLabV3+, and based on patch extraction using (**g**) FCN, (**h**) SegNet (**i**) U-Net and (**j**) DeepLabV3+.

Similar trends of performance are seen in the 2-class labeled UKMMC dataset, as given in Table 2. Here, the ground truth mask is only available for the whole prostate gland (WG). Again, the best DSC value of 91.9%, is generated by DeepLabV3+. Overall, the average improvement of the patch-wise method over without patch is by 8.7%. For qualitative analysis, visual inspection on the segmented image as shown in Figure 7 is performed. From Figure 7, it is clear that DeepLabV3+ is better than FCN, SegNet, and U-Net in segmenting the WG. Notably, FCN, SegNet, and U-Net are not able to accurately segment the WG, especially around the upper and lower sections of the WG.

To further confirm the better performance of patch-wise DeepLabV3+ over three other CNNs, a significance test is performed using the Wilcoxon signed-rank test generating *p*-values for the DeepLabV3+ versus the rest. The significance level is set at 0.05 and a null hypothesis is that the DeepLabV3+ versus three other CNNs will not improve the DSC values of the segmented images. The *p*-values tabulated in Table 3 for both NCI-ISBI 2013 and UKMMC datasets are all less than 0.05 which imply that we can reject the null hypothesis. In other words, this gives the indication that patch-wise DeepLabV3+ can better perform prostate segmentation than U-Net, SegNet, and FCN when measured in terms of DSC values.

The better performance of the DeepLabV3+ can be attributed to its architecture that uses the Xception model as the backbone and making use of atrous separable convolution for retaining the spatial resolution of the resultant feature maps. This allows the network to extract denser feature map by controlling the field view for precise localization and assimilation of context. In addition, utilizing atrous convolution enables the network to precisely define area with arbitrary scale by rebuilding them at various scales in atrous spatial pyramid pooling (ASPP). This has essentially resulted in a faster and efficient encoder–decoder network that covers better spatial information at multiple scales as compared to other encoder–decoder networks.

Performance comparison of DeepLabV3+ in terms of DSC values with some selected techniques including the state-of-the-art technique is summarized in Table 4. Direct comparison of DeepLabV3+ with other published techniques is difficult due to different in datasets, scanning protocols as well as numbers of images. Rough Comparison with other prostate segmentation techniques proposed in the past are listed in Table 4. If we consider the segmentation of WG by [19,20,30,31], the best DSC value is 89.4% by Yu et al. [31], which employed residual connection in U-Net. Our patch-wise DeepLabV3+ using a UKMMC dataset has resulted in a DSC value of 91.9%, which is 2.5% better than [31]. This may not be a completely fair comparison since it was not using the same dataset. However, this comparison may provide valuable insight on what is the best encoder–decoder segmentation method. Now, let’s consider segmentation of PZ and CG proposed by Litjens et al. [23] and Rundo et al. [32]. Then, comparing the DSC values indicate that our deep-wise DeepLabV3+ gave better DSC by 4.8% (PZ) and 3.7% (CG) than the multi atlas-based segmentation [23]. For more fair comparison, we compared our deep-wise DeepLabV3+ with the recently published state-of-the-art method proposed by Rundo et al. [32] which uses squeeze-and-excitation blocks in encoders and decoders of U-Net architecture. The deep-wise DeepLabV3+ gave a higher DSC value than the U-Net method in [32], with an increase of 2.9% (PZ) and 1.3% (CG).

## 5. Conclusions

In this paper, the evaluation of four encoder–decoder CNNs, including FCN, SegNet, U-Net, and DeepLabV3+ for segmentation of prostate T2W MRI is presented. The proposed methodology for the investigation includes methods for image pre-processing, patch extraction, data augmentation, and class weight balancing. These techniques have resulted in the normalized dataset, increased training images as well as its spatial variation, and reduced the effect of imbalance class. The selection of appropriate techniques prior to training the network is essential to achieve a well-trained network that gives good segmentation accuracy. Comparison with atlas-based, deformable model based, CNN-based, and state-of-the-art techniques has proven that patch-wise DeepLabV3+ gave better performance in segmentation of prostate WG, PZ, or CG. In summary, the best segmentation was achieved by a DeepLabV3+ model, which can be attributed to its atrous convolution operations and ASSP architecture. The accuracy can be further improved with more augmented data by injecting a generative adversarial network (GAN) to the deep models.

## Figures and Tables

**Table 1:** DSC values (in percentage) of prostate peripheral zone (PZ) and central gland (CG) segmentation by FCN, SegNet, U-Net, and DeepLabV3+ for test images from the NCI-ISBI 2013 dataset.

	Without Patch		Patch-Wise	
	PZ	CG	PZ	CG
FCN	69.2±5.8	85.1±4.8	72.7±5.1	88.6±2.9
SegNet	75.3±4.8	88.9±3.8	76.0±3.9	90.8±1.2
U-Net	76.3±3.5	89.8±2.6	76.8±3.3	91.6±1.4
DeepLabV3+	76.4±3.3	90.7±1.2	**78.9±1.9**	**92.8±0.7**
L. Rundo [32]	-	-	76.0±4.1	91.5±3.2

**Table 2:** DSC values (in percentage) of prostate whole gland (WG) segmentation by FCN, SegNet, U-Net, and DeepLabV3+ for test images from the UKMMC dataset.

	Without Patch	Patch-Wise
FCN	76.7±6.1	86.6±4.8
SegNet	79.8±5.7	84.3±4.2
U-Net	85.0±3.6	88.4±3.7
DeepLabV3+	83.2±4.7	91.9±2.0

**Table 3:** *P*-value of DeepLabV3+ versus U-Net, SegNet and FCN for NCI-ISBI-2013 and UKMMC datasets, using patch-wise implementation.

	NCI-ISBI 2013	UKMMC
DeepLabV3+ vs. U-Net	0.025	0.041
DeepLabv3+ vs. SegNet	0.022	0.046
DeepLabV3+ vs. FCN	0.016	0.035

**Table 4:** Comparison of DSC values (in percentage) of atlas-based, deformable model-based, CNN-based, and our patch-wise DeepLabV3+.

Work	Images	Dataset	Network	DSC (WG) or (PZ, CG)
Klein et al. 2008 [20]	50 volumes	3T MRI	Atlas matching using localized information	84.4
Guo et al. 2015 [19]	66 images	University of Chicago Hospital	Deformable model using SSAE	87.1
Litjen et al. 2012 [23]	48 images	3T MRI	Multi atlas-based segmentation	74, 89
Milletari et al. 2016 [30]	80 volumes	PROMISE12	V-Net	86.9
Yu et al. 2015 [31]	80 volumes	PROMISE12	ConvNet with residual connections	89.4
Rundo et al. 2019 [32]	40 images	NCI-ISBI 2013	U-Net with SE block	76.0±4.1, 91.5±3.2
**Our Work**	**40 images**	**NCI-ISBI 2013**	**Patch-wise DeepLabV3+**	**78.9±1.9**, **92.8±0.7**
**Our Work**	**11 images**	**UKMMC**	**Patch-wise DeepLabV3+**	**91.9±2.0**
